# Prevalence and characterization of plasmids carrying sulfonamide resistance genes among *Escherichia coli *from pigs, pig carcasses and human

**DOI:** 10.1186/1751-0147-52-47

**Published:** 2010-07-30

**Authors:** Shuyu Wu, Anders Dalsgaard, Anette M Hammerum, Lone J Porsbo, Lars B Jensen

**Affiliations:** 1 Department of Microbiology and Risk Assessment, National Food Institute, Technical University of Denmark, Mørkhøj Bygade 19, DK-2860 Søborg, Denmark; 2 Department of Veterinary Disease Biology, Faculty of Life Sciences, University of Copenhagen, Stigbøjlen 4, DK-1870 Frederksberg C, Denmark; 3 National Center for Antimicrobials and Infection Control, Statens Serum Institut, 5 Artillerivej, DK-2300 Copenhagen S, Denmark; 4 Shuyu Wu's present address changed to: US-CDC China Office, Suite 403, Dongwai Diplomatic Building, 23 Dongzhimenwai Dajie, Beijing 100600, China

## Abstract

**Background:**

Sulfonamide resistance is very common in *Escherichia coli*. The aim of this study was to characterize plasmids carrying sulfonamide resistance genes (*sul1*, *sul2 *and *sul3*) in *E. coli *isolated from pigs and humans with a specific objective to assess the genetic diversity of plasmids involved in the mobility of *sul *genes.

**Methods:**

A total of 501 *E. coli *isolates from pig feces, pig carcasses and human stools were tested for their susceptibility to selected antimicrobial. Multiplex PCR was conducted to detect the presence of three *sul *genes among the sulfonamide-resistant *E. coli *isolates. Fifty-seven sulfonamide-resistant *E. coli *were selected based on presence of *sul *resistance genes and subjected to conjugation and/or transformation experiments. S1 nuclease digestion followed by pulsed-field gel electrophoresis was used to visualize and determine the size of plasmids. Plasmids carrying *sul *genes were characterized by PCR-based replicon typing to allow a comparison of the types of *sul *genes, the reservoir and plasmid present.

**Results:**

A total of 109/501 isolates exhibited sulfonamide resistance. The relative prevalences of s*ul *genes from the three reservoirs (pigs, pig carcasses and humans) were 65%, 45% and 12% for *sul2*, *sul1*, and *sul3*, respectively. Transfer of resistance through conjugation was observed in 42/57 isolates. Resistances to streptomycin, ampicillin and trimethoprim were co-transferred in most strains. Class 1 integrons were present in 80% of s*ul1*-carrying plasmids and 100% of *sul3*-carrying plasmids, but only in 5% of *sul2*-carrying plasmids. The *sul *plasmids ranged from 33 to 160-kb in size and belonged to nine different incompatibility (Inc) groups: FII, FIB, I1, FIA, B/O, FIC, N, HI1 and X1. IncFII was the dominant type in *sul2*-carrying plasmids (52%), while IncI1 was the most common type in *sul1 *and *sul3*-carrying plasmids (33% and 45%, respectively). Multireplicons were found associated with all three *sul *genes.

**Conclusions:**

*Sul *genes were distributed widely in *E. coli *isolated from pigs and humans with *sul2 *being most prevalent. *Sul*-carrying plasmids belonged to diverse replicon types, but most of detected plasmids were conjugative enabling horizontal transfer. IncFII seems to be the dominant replicon type in *sul2*-carrying plasmids from all three sources.

## Background

High prevalence of sulfonamide resistance has been observed in Gram-negative bacteria from animals and humans all over the world [1-5]. In Denmark, sulfonamides are one of the most frequently used antimicrobials to treat diseases in pigs and sulfonamide resistance prevalence in *Escherichia coli *is relatively high along the food chain from pigs to pork and humans [3,5,6]. In contrast to the many different resistance genes described for other classes of antimicrobials, so far there are only three genes (*sul1*, *sul2 *and *sul3*) which have been identified encoding sulfonamide resistance [4,7]. *Sul1 *has almost exclusively been found on large conjugative plasmids and on class 1 integrons [3,6,8,9]. *Sul2 *was previously considered to be located on small non-conjugative plasmids, but recently the gene has also been found on a wide range of large conjugative plasmids [1,8] and have been linked to the prevalence of streptomycin resistance [3]. *Sul3 *was originally described from pigs in Switzerland in 2003 and has since been reported in animals as well as humans in many countries. A recent publication has linked *sul3 *to non-classic class 1 integrons [10]. However, knowledge about the structure and circulation of plasmids carrying *sul3 *has yet not been well described yet [7].

Genetic localization of *sul *genes on efficient mobile genetic structures probably contribute to the wide spread of sulfonamides resistance. Plasmids unavoidably play an important role in carrying and mobilizing *sul *genes [8]. The identification of plasmids associated with specific *sul *genes therefore helps understanding the mobilization capability of *sul*-genes among different bacterial species and enables determination of how sulfonamide resistance disseminates in different environments. Currently little is known about plasmid backbones associated with various *sul *genes especially in different reservoirs, e.g. animals and human [1,9]. The aims of this study were therefore to investigate the prevalence of the sulfonamide resistance genes (*sul1, sul2 *and *sul3*) in *E. coli *through the food chain from pigs to slaughter (pig carcasses) and humans and to assess the genetic diversity of plasmid, based on size and replicon, involved in the mobility of various *sul *genes from the three sources.

## Methods

### Bacterial strain collection

A total of 501 *E. coli *isolates collected from three sources were included in this study: 150 isolates were obtained in 2007 from fecal samples of Danish pigs as part of the Danish Integrated Antimicrobial Resistance Monitoring and Research Program (DANMAP) [5]; 275 isolates were obtained from pig carcasses in a Danish pig slaughterhouse in November 2007 [11]; and 76 isolates were obtained from stool samples of Danish healthy humans between May and June 2008, of which only two had been travelling abroad within three months before sampling [12].

Sampling and strain isolation for *E. coli *from pig feces has been described previously [5,11,12]. Only one isolate was collected from each pig fecal and human stool sample. Multiple isolates were collected from each pig carcass sample, but only representative isolates with distinct pulsed-field gel electrophoresis (PFGE) patterns were included in the characterization of plasmids.

### Antimicrobial susceptibility testing

Antimicrobial resistance profiles for all isolates were determined by an automated microdilution method (Trek Diagnostic Systems, East Grinstead, UK) for 17 antimicrobial agents included in DANMAP [5]. The results were interpreted in accordance with EUCAST guidelines [13], where the Minimum Inhibitory Concentration (MIC) breakpoint for sulfamethoxazole resistance is > 256 mg/L.

### Detection of sul genes and integrons

Multiplex PCR was performed on boiled cell lysates of the sulfonamide-resistant isolates. Primers developed by Kerrn *et al. *[14] were used for detection of *sul1 *and *sul2 *and a new primer-set for *sul3 *was designed for this study (*sul3*-F: 5'-CAGATAAGGCAATTGAGCATGCTCTGC-3', *sul3*-R: 5'-AGAATGATTTCCGTGACACTGCAATCATT-3'). PCR was performed in a 20 μL mixture, including 2 μL template DNA, 3 μL 10× reaction buffer (Ampliqon, Herlev, Denmark); 1 μL MgCl_2 _(25 mM; Ampliqon); 0.25 μL dNTPs; 0.25 μL Taq Polymerase (50 mM, Ampliqon); 2.5 μL forward and reverse primer mixture (volume for *sul1*:*sul2*:*sul3 *= 1:1:2). Amplification was carried out by heating for 5 min at 94°C, followed by 30 cycles at 94°C for 1 min, 68°C for 1 min and 72°C for 2 min, followed by 72°C for 10 min. The sizes of the amplicon were 433 bp for *sul1*, 293 bp for *sul2 *and 569 bp for *sul3*. Presences of class 1 and class 2 integrons were detected by primers for integrase 1 and integrase 2 described previously [14,15]. Positive and negative controls were included in all PCR arrays.

### Plasmid-mediated sulfonamide resistance transferability

A proportion of sulfonamide-resistant *E. coli *isolates (18 from humans, 18 from pig feces and 21 from pig carcasses) representing different *sul *genes were selected for further plasmid characterization. Conjugation experiments were performed at 37°C by filter mating assay using *E. coli *K12-J53 (nalidixic acid-resistant) as recipient and transconjugants were selected on Mueller-Hinton agar plates supplemented 256 mg/L sulfamethoxazole and 40 mg/L nalidixic acid.

When donor strains were resistant to nalidixic acid or resistance plasmid transfer failed in the mating experiments or when plasmid co-transfer occurred, electroporation was done to obtain recipients with single plasmid carrying *sul *genes. Plasmid DNA was purified by the QIAGEN Plasmid Mini kit (Qiagen, Hilden, Germany) as described by the manufacturer. Purified plasmids were used to transform ElectroMAX™DH10B™competent cells (Invitrogen, Paisley, United Kingdom) using a Bio-Rad MicroPulser (Bio-Rad Laboratories, CA, USA) under standard conditions (1.8 kv, 180 Ω and 25 μF). Transformants were selected on Mueller-Hinton agar plates supplemented with 256 mg/L sulfamethoxazole and confirmed to harbor corresponding *sul *genes by multiplex PCR assay as described earlier.

### S1 nuclease digestion of plasmids and PFGE

S1 nuclease digestion followed by PFGE was performed to visualize and determine the molecular size of plasmids [14]. Agarose plugs were prepared according to the *PulseNet *protocol [16] except the concentration of the cells was adjusted to 0.83-0.85 on a Dade Micoscan turbidity meter (Dade Behring, CA, USA). Slices of plugs were digested with 5 U of S1 nuclease (Promega, Madison, WI, USA) for 45 min at 37°C. The plasmids were separated in a 1% SeaKem Gold agarose (Cambrex, East Rutherford, NJ, USA) gel using the CHEF DR III System (Bio-Rad, Hercules, CA) in 0.5× Tris-borate EDTA at 14°C and an angle of 120 at 6 V/cm gradient with pulse ramping from 6.8 to 38.4 s over 18 h.

### Plasmid replicon type determination

Plasmids from parental and transformant/transconjugant strains were assigned to 18 incompatibility (Inc) groups by PCR-based replicon typing (PBRT) of total DNA using previously described primers and conditions [17]. IncQ was screened as described previously [18]. The presence of IncX1 was investigated using the primers (IncX1-F: 5'-GCAGATTGATTCACGTGAAG-3', IncX1-R 5'-CCTCTGAAACCGTATGGTATTC-3'). All positive results generated by multiplex PCR were confirmed using single primer pair.

## Results and discussion

### Prevalence of sulfonamide resistant E. coli and sul genes

A total of 109 sulfonamide resistant *E. coli *isolates were detected and prevalences of sulfonamide resistance were 17% for pig feces (26/150), 18% from pig carcasses (49/275) and 45% from humans stool (34/76) (Table 1). The prevalence of *sul1 *gene varied from 23% to 29% in *E. coli *from the three sources. The *sul2 *gene was the most prevalent sulfonamide resistance gene found in all of the three sources (> 40%). The *sul3 *gene was present alone in 9% of pig feces and 10% of pig carcasses, while in human isolates it was detected simultaneously with *sul2 *in two *E. coli *isolates. The presence of two *sul *genes was detected in *E. coli *from all sources (18% from pig feces, 20% from pig carcasses and 31% from humans).

**Table 1:** Prevalence of *sul1*, *sul2 *and *sul3 *genes in sulfonamide-resistant *E. coli *isolated from pig feces, pig carcasses and human stools

Origin	No. of isolates tested	No. of isolates with *sul *genes (percentage)			
		
		*sul1 *only	*sul2 *only	*sul3 *only	two *sul *genes
Human stool	26	6 (23%)	12 (46%)	0	8 (31%)^a^
Pig carcass	49	13 (27%)	21 (43%)	5 (10%)	10 (20%)^b^
Pig feces	34	10 (29%)	15 (44%)	3 (9%)	6 (18%)^c^

^a ^Six strains were positive for both *sul1 *and *sul2*; two strains contained both *sul2 *and *sul3*.

^b ^Seven strains contained both *sul1 *and *sul2*; one strain contained *sul1 *and *sul3*; and two strains harbored *sul2 *and *sul3*.

^c ^Six strains contained both *sul1 *and *sul2*.

A higher prevalence of *sul2 *than *sul1 *and *sul3 *in *E. coli *from animals and humans has been observed in previous studies in Denmark as well as in other countries [3,6,14,19,20]. In the UK, sulfonamide resistance in human *E. coli *persists undiminished with *sul2 *maintaining dominant despite that the human usage of sulfonamides had been terminated decades ago[1,2]. We found that *sul3 *occurred with the lowest prevalence of the three *sul *genes among all *E. coli *from the three sources, which corresponds to previous observations [3,6,9,21,22]. The higher prevalence of combined *sul *genes in human *E. coli *isolates compared with food animal isolates was also observed in earlier studies [3,6]. This is likely due to the acquisition of additional genes from different sources (animals, food and environment), as well as the selective pressure by the human's consumption of antimicrobial agents.

Sulfonamides are widely used in Danish veterinary practice. Combined formulations of sulfonamide/trimethoprim have been frequently used in Danish pig production [5]. This may explain why sulfonamide resistance occurs with high prevalence in *E. coli *isolates from Danish pigs. From the previous epidemiological study of the human isolates included in this investigation no strong correlation between prevalence of sulfonamide resistance in isolates of human origin and human usage of sulfonamides could be found.

### Transferability of sul genes

A total of 57 sulfonamide-resistant *E. coli *isolates (18 from pig feces, 21 from pig carcasses and 18 from humans) were included for conjugation assay and plasmid analysis. Conjugative plasmids were successfully transferred from 42 isolates (82%), including 22 isolates where the *sul *plasmids were co-transferred with another plasmid (data not shown). Electro-transformation was conducted to ensure transconjugants containing only one plasmids encoding for *sul*-resistance. This was performed for the 22 isolates containing multiple conjugative plasmids, including 9 *E. coli *isolates with non-conjugative plasmids and 6 isolates showing resistance to nalidixic acid. The results showed, that 53/57 transconjugants/transformants obtained a single *sul *gene, while the remaining four isolates (all from humans) contained single plasmids of different sizes carrying both *sul1 *and *sul2 *genes (Table 2).

**Table 2:** Characterization of *sul *genes and plasmids in 57 *E. coli* isolates.

*sul *gene located on plasmids	Strain ID	Sample source	Inc group	Plasmid size (kb)	Co-transferred resistance	Conjugative	Integrase
*sul1*	70-7-3	pig feces	I1	60	TET TMP	Yes	*IntI1*
*sul1*	70-10-1	pig feces	I1	78	STR SPE TET	Yes	*IntI1*
*sul1*	70-13-2	pig feces	I1	140	STR SPE	Yes	*IntI1*
*sul1*	70-58-1	pig feces	I1	78	SPE TET	Yes	*IntI1*
*sul1*	v3-s1-8-4	pig carcass	I1	78	TET TMP	Yes	*IntI1*
*sul1*	v2-f2-2	pig carcass	N	33	TET TMP	Yes	*IntI1*
*sul1*	v2-s3-3-10	pig carcass	N	33	TET TMP	Yes	*IntI1*
*sul1 *	v2-s3-2-3	pig carcass	FIB	105	SPE STR	Yes	*IntI1*
*sul1*	3847	human stool	FIB	80	AMP SPE STR	Yes	*IntI1*
*sul1*	v4-s1-20-3	pig carcass	HI1	160	SPE STR TET	Yes	*IntI1*
*sul1*	70-7-2	pig feces	FII	45	AMP	Yes	
*sul1*	3796	human stool	FIA, FIB	95	AMP AUG GEN TMP	No	
*sul1*	3880	human stool	FIA, FIB	78	TMP	No	
*sul1*	3849	human stool	FIB, FII	80	STR	No	
*sul1*	3846	human stool	B/O	55	AMP AUG	Yes	
*sul1*	70-138-3	pig feces	Unknown	85	STR TMP	No	*IntI1*
*sul1*	3806	human stool	Unknown	40	AMP TET TMP	Yes	*IntI1*

*sul2*	70-13-7	pig feces	FII	55	AMP	Yes	
*sul2*	70-165-18	pig feces	FII	45	AMP	Yes	
*sul2*	70-13-9	pig feces	FII	55	AMP STR	Yes	
*sul2*	70-56-3	pig feces	FII	55	AMP STR TMP	Yes	
*sul2*	70-58-2	pig feces	FII	40	AMP STR TMP	Yes	
*sul2*	v2-f3-2	pig carcass	FII	50	AMP	Yes	
*sul2*	v2-s1-3-5	pig carcass	FII	45	AMP STR	Yes	
*sul2*	v3-s1-2-8	pig carcass	FII	55	AMP STR	Yes	
*sul2*	v4-s3-17-3	pig carcass	FII	40	AMP STR	Yes	
*sul2*	v2-f3-6	pig carcass	FII	55	AMP STR TET TMP	Yes	
*sul2*	v2-s1-1-10	pig carcass	FII	55	AMP GEN STR TMP	Yes	
*sul2*	3783	human stool	FII	50	AMP TMP	--^a^	
*sul2*	3816	human stool	FII	33	AMP STR TET TMP	No	
*sul2*	3837	human stool	FII	33	AMP STR TET TMP	No	
*sul2*	3859	human stool	FII	55	STR	--	
*sul2*	70-141-6	pig feces	FIB	120	TET	Yes	
*sul2*	70-166-11	pig feces	B/O	80	TMP	Yes	*IntI2*
*sul2*	70-10-6	pig feces	B/O	70	AMP STR TET	Yes	*IntI1*
*sul2*	70-11-5	pig feces	B/O	78	AMP STR SPE TMP	Yes	
*sul2*	3841	human stool	B/O	55	AMP STR	Yes	
*sul2*	v2-s1-1-8	pig carcass	I1	50	AMP SPE STR TMP	--	
*sul2*	3834	human stool	FIA, FIB	60	AMP GEN STR TET TMP	--	*IntI1*
*sul2*	v2-s3-1-8	pig carcass	Unknown	45	AMP STR	Yes	
*sul2*	3815	human stool	Unknown	80	AMP SPE STR	Yes	*IntI1*
*sul2*	3810	human stool	Unknown	95	STR	--	*IntI1*

*sul1+2*	3853	human stool	FIB	130	AMP CHL STR TET TMP	No	*IntI1*
*sul1+2*	3823	human stool	FIB	139	AMP STR TMP	--	
*sul1+2*	3872	human stool	FIB, FII	100	STR TET TMP	No	
*sul1+2*	3881	human stool	FIA, FIB, FII	110	GEN STR TET TMP	No	

*sul3*	70-13-1	pig feces	I1	80	CHL	Yes	*IntI1*
*sul3*	70-11-1	pig feces	I1	78	----	Yes	*IntI1*
*sul3*	v2-s3-3-6	pig carcass	I1	78	----	Yes	*IntI1*
*sul3 *	v2-s3-2-4	pig carcass	I1	78	CHL SPE STR TMP	Yes	*IntI1*
*sul3*	v4-s3-3-4	pig carcass	I1	55	CHL	Yes	*IntI1*
*sul3 *	V3-s3-2-4	pig carcass	X1	115	AMP NEO SPE TMP	Yes	*IntI1*
*sul3*	v4-f1-2	pig carcass	FII	78	CHL	Yes	*IntI1*
*sul3*	v4-s1-20-4	pig carcass	FIB, FIC	65	SPE	Yes	*IntI1*
*sul3*	70-8-1	pig feces	FIB, FIC	78	SPE	Yes	*IntI1*
*sul3*	v4-s1-20-6	pig carcass	Unknown	55	CHL	Yes	*IntI1*
*sul3*	v2-s1-1-9	pig carcass	Unknown	33	CHL	Yes	*IntI1*

AMP: ampicillin; AUG: amoxillin + clavulanat; CHL: chloramphenicol GEN: gentamicin; SPE: spectinomycin; STR: streptomycin; NEO: neomyxin; SMX: sulfamethoxazole; TET: tetracycline; TMP: trimethoprim.

^a ^conjugation not conducted.

Additional phenotypically expressed resistances were co-transferred with sulfonamide resistance by 55 plasmids (96%), resulting in diverse resistance patterns (Table 2) indicating possible co-selection for all three tested sulfonamide resistance genes. Overall, the most frequently co-transferred resistances were to streptomycin (54%), ampicillin (51%) and trimethoprim (46%). These three resistances were co-transferred more frequently by *sul2 *plasmids than those by *sul1 *and *sul3 *plasmids with highest frequency for streptomycin and ampicillin resistance (both 79%), indicating that the use of these antimicrobials possibly contribute to the predominance of the *sul2 *gene via co-selection, which corroborates previous studies by Bean *et al. *[1,2]. Tetracycline resistance was co-transferred in 16 plasmids carrying *sul1 *and/or *sul2 *genes. Chloramphenicol resistance was found to be co-transferred in 7 plasmids with 6 of them being *sul3 *associated. The strong link between chloramphenicol resistance and *sul3 *presence has already been reported for *Salmonella *and *E. coli *[9,23].

The presence of *intI1 *was detected in *sul1 *(71%), *sul2 *(17%) and *sul3 *(100%) -carrying plasmids from all three sample reservoirs (Table 2). Two out of 28 *intI1*-carrying plasmids could not be transferred by conjugation. Only one of 57 isolates carrying the *sul2 *plasmid contained *intI2*. It is interesting to note, that all *sul3 *plasmids were conjugative and harbored *intI1 *as recently detected [9]. The association of *sul3*-carrying conjugative plasmids with integrons in *E. coli *isolates from pigs (carcass or feces) could probably facilitate the further spread of this gene to other bacteria and reservoirs via food chain.

### Plasmid analysis and replicon typing

S1 nuclease digestion followed by PFGE showed that the 15 plasmids carrying single *sul1 *genes ranged from 33-160 kb in size. Replicon typing demonstrated seven different incompatibility groups (I1, N, FIB, FIA, HI1, FII and B/O) for the plasmids (Table 2). IncI1 was the most common group, accounting for 5 *intI1*-containing conjugative plasmids (from pig feces and carcasses). Out of the six *sul1 *plasmids originating from human specimens, four belonged to FI or FII type (three plasmids had multireplicons FIA-FIB or FII-FIB), one plasmid belonged to B/O and one plasmid could not be assigned. This replicon profile in human *E. coli *isolates seems different from those found in *E. coli *from pigs which mostly belonged to I1 and N. Due to the limited number of strains obtained from each source, a firm conclusion on whether specific replicon profiles are associated with pigs or humans would require the testing of additional strains.

The 25 *sul2*-carrying plasmids ranged from 33-120 kb in size. Five different incompatibility groups (FII, B/O, FIB, I1 and FIA) were demonstrated (Table 2). Among them FII was the predominant replicon type (52%) and distributed equally in *E. coli *from the three sources. This is in contrast to a study in the UK where the FII replicon was not detected in 33 *sul2*-carrying plasmids in *E. coli *isolated from hospitals [1]. The 15 FII plasmids were similar in size (33-55 kb) and none contained *intI1*. However, the plasmids were associated with various resistance phenotypes and conjugation capabilities. It is possible that they represent the same plasmid backbone circulating in pigs and humans. The B/O replicon type was found in plasmids from *E. coli *isolated from both human and pig, but with different resistance profiles, indicating they are different plasmids sharing the same backbone. FIA, FIB, and I1 replicons were also found to be associated with *sul2 *genes. Three *sul2*-carrying plasmids were negative for all the tested Inc groups. For the four plasmids carrying both *sul1 *and *sul2 *genes, two harbored the FIB replicon alone, one harbored FIB-FII and one had FIA-FIB-FII multireplicons.

Overall, IncF (FIA, FIB, FIC and FII) seemed to be the most common replicon associated with *sul2*, accounting for 72% *sul2*-carrying plasmids (21/29). IncF plasmids, which are considered having a narrow host range and being conjugative, seemed to be well adapted to *E. coli *as it was found in more than 50% of *E. coli *strains from different sources (especially FIB and FII) [24,25]. Localization on common plasmids such as IncF could indicate transmission between the three reservoirs studied.

The 11 *sul3*-carrying plasmids ranged from 33-115 kb in size. Replicon typing detected five different incompatibility groups: I1, FII, FIB, FIC and X1 (Table 2). IncI1 was found to be the most prevalent (45%) and all were conjugative and associated with class 1 integrons. The IncI1 plasmids had similar sizes, but differed in the resistance profiles. FII, X1 and FIB-FIC were also found to be associated with the *sul3 *gene. Two *sul3*-carrying plasmids were negative for all the tested Inc groups.

When comparing plasmid size and the resistance phenotype, we found that *E. coli *isolates with larger sized plasmids did not always show resistance to a higher number of different antimicrobials. For instance, the 120 kb-sized plasmid contained in isolate 70-141-6 was only associated with resistance to tetracycline (Table 2). However, isolates with smaller plasmids of only 33-kb size (isolates 3816 and 3837) showed resistance to ampicillin, streptomycin, tetracycline and trimethoprim. This indicates that resistance genes only account for a small part of the plasmid gene sequence and more studies are needed to determine the contents and function of such plasmids.

The findings of diverse incompatibility groups among various *sul *genes indicate that *sul *genes have been able to transfer into multiple plasmid backbones on multiple occasions, or that the plasmid backbones have diversified extensively since the acquisition of *sul *genes [1]. The IncF in our study was the most prevalent, carrying FII alone or in combination with FIA or/and FIB or/and FIC (multireplicon). This suggests that these plasmids are evolving through replicon sequence divergence, mosaicism and replicon co-integration in resolution process [26]. As human stays on the top of the food chain, the *sul *plasmids from healthy human reflects a more mixed population compared with pig or pork (partly represented by pig carcasses) also reflecting other reservoirs that connected to the farm to fork chain.

*Sul*-carrying plasmids in pig feces and on pig carcasses represent a pool of resistance genes that may transfer to human via the food chain, the most important non-human reservoir for transmission of antimicrobial resistance to humans. In fact, the colonization of sulfonamide-resistant *E. coli *from animal sources (chicken or pig) into the human gut has been successfully demonstrated in both *in vitro *and *in vivo *studies [27,28]. The transfer of the *sul2 *gene from an *E. coli *strain of a pig origin to a sulfonamide-sensitive *E. coli *strain of human origin was demonstrated in the intestine of mice and transfer of *sul2 *has also been detected among *E. coli *in the human intestine [15]. By combining all these date it could be speculated that consumption of pork contaminated with sulfonamide-resistant *E. coli *could result in the transfer of *sul *genes from pigs to humans.

## Conclusions

To our knowledge, this is the first study to assess the plasmid replicons involved in various *sul *genes from different reservoirs. The diverse replicon profiles indicate no clear association between the replicon types and specific *sul *genes or sample sources. However, the localization of *sul *genes on wide spread replicons such as IncF is very likely to contribute to the dissemination of sulfonamide resistance. In addition, all *sul3*-carrying plasmids were found to be conjugative and associated with class 1 integrons. This underscores the potential of *sul3 *to become more widespread in the future.

## Competing interests

The authors declare that they have no competing interests.

## Authors' contributions

WS performed the experiment and was responsible for the data analysis and writing the manuscript. AD and LJE were involved in the study design and preparation of the manuscript. AMH and LJP participated in the study design and data analysis. All authors read and approved the final manuscript.
